# Antipyretic, analgesic and anti-inflammatory activity of *Viola betonicifolia* whole plant

**DOI:** 10.1186/1472-6882-12-59

**Published:** 2012-05-02

**Authors:** Naveed Muhammad, Muhammad Saeed, Haroon Khan

**Affiliations:** 1 Department of Pharmacy University of Peshawar, 25120, Peshawar, Pakistan; 2 Gandhara College of Pharmacy, Gandhara University, Peshawar, Pakistan

**Keywords:** *Viola betonicifolia*, Antipyretic, Analgesic, Anti-inflammatory

## Abstract

**Background:**

Pyrexia, algesia and inflammation are associated with several pathological conditions. Synthetic drugs available for the treatment of these conditions cause multiple unwanted effects. Several studies are ongoing worldwide to find natural healing agents with better safety profile. The current study was thus aimed at evaluating antipyretic, analgesic and anti-inflammatory activities of the methanolic extract of whole plant of *V. betonicifolia* (VBME).

**Methods:**

VBME was employed to assess antipyretic activity in yeast induced hyperthermia. Analgesic profile was ascertained in acetic acid induced writhing, hot plat and tail immersion test. Nevertheless, the anti-inflammatory activity was tested in carrageenan induced paw edema and histamine induced inflammatory tests. BALB/c mice were used at test doses of 100, 200 and 300mg/kg body weight intra peritoneally (i.p).

**Results:**

In yeast induced pyrexia, VBME demonstrated dose dependently (78.23%) protection at 300mg/kg, similar to standard drug, paracetamol (90%) at 150mg/kg i.p. VBME showed a dose dependent analgesia in various pain models i.e. acetic acid, hot plat and tail immersion having 78.90%, 69.96% and 68.58% protection respectively at 300mg/kg. However, the analgesic action of VBME was completely antagonized by the injection of naloxone like opiate antagonists. Similarly carrageenan and histamine induces inflammation was significantly antagonized by VBME, 66.30% and 60.80% respectively at 300mg/kg.

**Conclusions:**

It is concluded that VBME has marked antipyretic, analgesic and anti-inflammatory activities in various animal models and this strongly supports the ethnopharmacological uses of *Viola betonicifolia* as antipyretic, analgesic and anti-inflammatory plant.

## Background

Over the centuries, phytopharmaceuticals have been utilized by different communities of the world [1]. The local communities of different regions of Pakistan have been using medicinal plants as a primary source of their health care system and infact these medicinal plants are used to cure a large number of diseases [2]. In Pakistan, this trend is well established in the name of Hikmat/Tabib and approximately, 600–1000 medicinal plants of the country have been used in the management of different pathological conditions by more than 40,000 registered and a large number of unregistered Hakims or Tabib [3]. This practice is based on experiences, without any scientific evidence and therefore, need proper validation on scientific grounds [4].

*V. betonicifolia* belongs to family Violaceace locally which is known as banafsha. *V.betonicifolia* is found naturally in various countries of the world like Pakistan, India, Nepal, Srilanka, China, Malaysia and Australia. In Pakistan, it is available in Swat, Hazara and Dir districts. The folk use of this plant is antipyretic, astringent, diaphoretic, anticancer and purgative. It has been used in the treatment of various neurological disorders including epilepsy and insomnia [5]. Additionally, it has been used in the treatment of sinusitis, skin and blood disorders and pharyngitis [6]. Roots are used for kidney diseases, pneumonia and bronchitis. Flowers are recommended for the treatment of asthma, cough and colds while leaves are useful for the treatment of boils [7]. Recently we have tested the crud methanolic as well as the subsequent solvent fraction of *V. betonicifolia* for various pharmacological activities [8,9]. The current study was designed to provide scientific evidence to the ethnobotanical uses of the plant in the treatment of pyrexia, algesia and inflammation in various animal models.

## Methods

### Chemicals

Paracetamol (Tianjin Bofa Pharmaceutical Co, Lit., China), Diclofenac sodium (Suzhou Ausun Chemical Co, Lit.,China), Acetic acid, Brewer’s yeast (Merck Germany), Carrageenan (Sigma Lambda, USA), Histamine (Alfa Aesar - A Johnson Matthey Company), Naloxone (Acent Scientific Company), Tramadol^R^ (Searle Pakistan Ltd.). Sterile normal saline was used in all experiments as control while methanolic extract was prepared in normal saline.

### Animals

BALB/c mice of either sex were used in all experiments. Animals were purchased from the Pharmacology Section of the Department of Pharmacy, University of Peshawar, Peshawar, Pakistan. The animals were maintained in standard laboratory conditions (25°C and light/dark cycles i.e. 12/12h) and were fed with standard food and water *ad libitum*. The experimental protocols were approved by the ethical committee of the Pharmacy Department, University of Peshawar, Peshawar, Pakistan.

### Plant material

Whole plant of *V. betonicifolia* was collected from Swat, Khyber Pakhtunkhawa, Pakistan, in April 2010. Plant specimen was identified by Taxonomist, Department of Botany, University of Peshawar and a specimen was deposited there in the herbarium with voucher number 6410/Bot. The collected whole plant (12kg) was air dried and powder. The powder was extracted by maceration with methanol at room temperature for 14days with occasional shaking. The methanolic extract was filtered and concentrated under vacuum using rotary evaporator at low temperature (45°C). The methanolic extract was dissolved in distilled water and further fractionated with chloroform, *n*-hexane, ethyl acetate, *n*-butanol and aqueous fractions.

### Acute toxicity

The acute toxicity test was carried out for VBME to evaluate any possible toxicity. BALB/c mice (n = 6) of either sex were treated with different doses (500, 1000 and 2000mg/kg, p.o.), while the control group received saline (10ml/kg). All the groups were observed for any gross effect for first 4h and then mortality was observed after 24h [10].

### Antipyretic test

The antipyretic activity was evaluated for VBME using BALB/c mice (25–30g) of either sex. The selected animals were healthy and were acclimatized to laboratory conditions before the start of experiment. The animals were divided into five groups each of six mice. The normal body temperature of each mouse was recorded using digital thermometer and then pyrexia was induced in all mice by injecting 20% aqueous suspension of Brewer’s yeast (10ml/kgs.c.). All groups were fasted overnight but allowed free accesses to drinking water and after 24h rectal temperature of each mouse was recorded. The induction of pyrexia was confirmed by rise in temperature more than 0.5°C, while animals showed rise in temperature less than 0.5°C were excluded from experiment [11]. Group I received saline (10ml/kg) as a negative control, Group II received paracetamol (150mg/kg) as a standard drug while the remaining groups III, IV and V received 100, 200 and 300mg/kg i.p. VBME respectively. After drugs administration, rectal temperature was again recorded periodically at 1, 2, 3, 4 and 5h of drugs administration. The percent reduction in pyrexia was calculated by the following formula.

Percent reduction = B – C_n_/B – A × 100

Where, B represents temperature after pyrexia induction; C_n_ temperature after 1, 2, 3, 4 and 5 h and A, normal body temperature. 

### Analgesic activity

#### Acetic acid induced writhing test

BALB/c mice of either sex (n = 6) weighing 18–22g were used. All animals were withdrawn from food 2h before the start of experiment and were divided in five groups. Group I was injected with normal saline (10ml/kg) as control, Group II received standard drug diclofenac sodium (10mg/kg) while the remaining groups III, IV and V were injected with 100, 200 and 300mg/kg i.p. of VBME respectively. After 30min of saline, diclofenac sodium and plant extract injection, the animals were treated i.p. with 1% acetic acid. The number of abdominal constrictions (writhes) were counted after 5min of acetic acid injection for the period of 10 min [10].

#### Hot plat test

BALB/c mice of either sex (n = 6) weighing 18–22g were acclimatized to laboratory conditions one hour before the start of experiment with food and water available *ad libitum*. Animals were then subjected to pre-testing on hot plat (Havard apparatus) maintained at 55 ± 0.1°C. Animals having latency time greater than 15 s on hot plate during pre-testing were rejected (latency time) [12]. All the animals were divided in eight groups each of six mice. Group I was treated with saline (10ml/kg), group II was treated with Tramadol^R^ (30mg/kg i.p). Group III, IV and V were treated with 100, 200 and 300mg/kg VBME, i.p. respectively. After 30min of treatment the animals were placed on hot plat and the latency time (time for which mouse remains on the hot plate (55 ± 0.1°C) without licking or flicking of hind limb or jumping) was measured in seconds. In order to prevent the tissue damage a cut-off time of 30 s were imposed for all animals. To find out the opiodergic mechanism in the analgesic activity of VBME, Groups VI and VII were treated with naloxone (0.5mg/kg s.c.) and after 10min these groups were treated with VBME (200 and 300mg/kg, i.p), while group VIII was treated with Tramadol^R^ (30mg/kg i.p.) after 10min of naloxone injection. The latency time for all groups was recorded at 0, 30, 60, 90 and 120min. Percent analgesia was calculated using the following formula:

% Analgesia = (Test latency – control latency)/(Cut – off time – control latency) × 100

#### Tail immersion test

BALB/c mice of either sex were divided into five groups each of six animals (18–22g). Saline (10ml/kg), VBME at the dose of 100, 200 and 300mg/kg, and Tramadol^R^ (30mg/kg) were administered intraperitoneally. The animal was kept in vertical position to hang the tail, which was up to 5cm into a pot of hot water maintained at 55 ± 0.5°C. The time in seconds to withdraw the tail out of water was taken as the reaction time (Ta). The reading was taken after 0, 30, 60, 90 and 120min of administration of the test drugs [13]. The cut-off time, i.e. time of no response was put at 30s, while Tb was consider the reaction time for control group.

Percentage analgesic activity = Ta – Tb/Tb × 100

### Anti-inflammatory activity

#### Carrageen induced paw edema

The anti-inflammatory activity was performed on mice of either sex (25–30g). The animals were randomly divided in five groups each of six animals [14]. Group I was treated with normal saline (10ml/kg), group II with diclofenac sodium (10mg/kg), rest of the groups were treated with VBME (100, 200, and 300mg/kg, i.p). After thirty minutes of the above intraperitoneal administration, carrageenan (1%, 0.05ml) was injected subcutaneously in the sub plantar tissue of the right hind paw of each mouse. The inflammation was measured using plethysmometer (LE 7500 plan lab S.L) immediately after injection of carrageenan and then 1,2,3,4 and 5h. The average foot swelling in drug treated animal as well as standard was compared with that of control and the percent inhibition (anti-inflammatory activity) of edema was determined using the formula.

Percent inhibition = A-B/A × 100, where A represent edema volume of control and B as paw edema of tested group.

#### Histamine induced paw edema

Animals were divided as in the previous experiment and inflammation was induced by subcutaneous injection of 0.1ml of freshly prepared solutions of histamine (1mg/ml) into the hind paws of the mice [13]. The percent inhibition of paw edema induced by each test sample was calculated as described in case the carrageenan induced paw test.

### Phytochemical status

Preliminary phytochemical tests were performed for VBME. The presence of alkaloid content was determined by performing Mayer’s test; white precipitate (ppt) indicated the presence of alkaloids [15]. Flavonoids were determined when addition of few drops of sodium hydroxide solution, formed intense yellow coloration that became colorless after addition of dilute acetic acid. Saponins were identified by formation of froth upon simple shaking (frothing test). Tannins and phenols were identified on addition of ferric chloride to the extract solution; the appearance of blue or green ppt indicated the presence of tannins [16]. Sterols and triterpenoids were identified on addition of few drops of acetic anhydride to the extract solution, boiled, cooled and then add concentrated sulphuric acid, producing brown ring at the junction of two layers, the turning of upper layer to green indicated sterols and deep red color indicated triterpenoids [17].

### Statistical analysis

The results obtained were expressed as mean ± SEM (Standard error of mean) of six animals. For statistical analysis, ANOVA was followed by post hoc Dunnett’s test for multiple comparisons. Effects were considered to be significant at the *P* < 0.05 level.

## Results

### Acute toxicity

VBME was found safe at all test doses (500, 1000 and 2000mg/kgi.p.). During 24h assessment time, test animals were found normal.

### Antipyretic test

**Figure 1:**
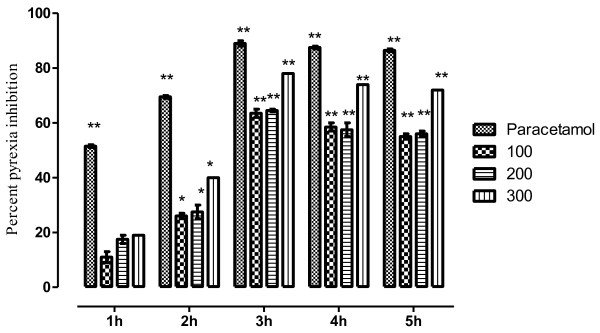
**Antipyretic effect of VBME in mice.** Bar presents the percent inhibition of pyrexia after 1,2,3,4 and 5h of the treatment with paracetamol (150mg/kg) and VBME (100–300mg/kg). The data was analyzed by ANOVA followed by Dunnett’s test. Asterisks indicated statistically significant values from control. *P < 0.05, **P < 0.01.

The VBME markedly (*P* < 0.01) attenuated hyperthermia induced by yeast. The inhibition was dose dependent and remained significant up to 3h of administration as shown in Table 1. The maximum antipyretic effect was observed at 300mg/kg i.e. 78.23% while, the antipyretic effect of paracetamol was 90%. The percent pyrexia inhibition is shown in Figure 1.

**Table 1:** Effect of VBME at 100, 200 and 300mg/kg i.p. in yeast induced pyrexia

**Treatment**	**Dosemg/kg**	**Rectal temperature (°C)**						
		**After administration of drug**						
		**Normal (A)**	**after 24h (B)**	**1h (C1)**	**2h (C2)**	**3h (C3)**	**4h (C4)**	**5h (C5)**
Saline	10mL	36.66 ± 0.11	38.92 ± 0.34	38.82 ± 0.21	38.78 ± 0.11	38.68 ± 0.20	38. 68 ± 0.20	38.72 ± 0.15
Paracetamol	150mg	37.08 ± 0.08	39.46 ± 0.04	38.20** ± 0.01	37.80** ± 0.03	37.30** ± 0.02	37.35 ** ± 0.28	37.38** ± 0.04
VBME	100mg	37.06 ± 0.02	39.70 ± 0.43	39.44 ± 0.22	38.98* ± 0.22	38.04** ± 0.11	38.16** ± 0.12	38.22** ± 0.11
	200	37.00 ± 0.14	39.00 ± 0.11	38.68 ± 0.02	38.40*s ± 0.25	37.72** ± 0.45	37.80** ± 0.11	37.85 ± 0.41
	300	37.02 ± 0.12	39.84 ± 0.20	39.30 ± 0.31	38.70* ± 0.12	37.64** ± 0.44	37.74** ± 0.10	37.80** ± 0.23

Data are reported as mean ± S.E.M. for group of six animals. The data was analyzed by ANOVA followed by Dunnett’s test. Asterisks indicated statistically significant values from control. *P < 0.05, **P < 0.01.

### Analgesic activity

#### Acetic acid induced test

**Figure 2:**
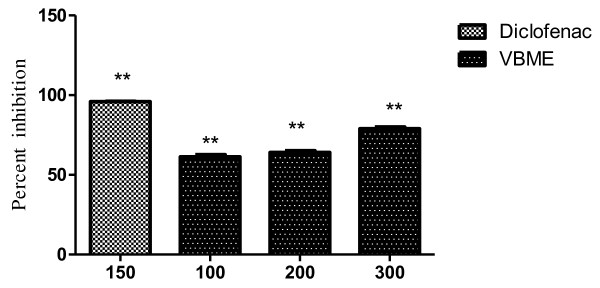
**Percent analgesic activity of VBME (100, 200 and 300mg/kg) in acetic acid induce pain model.** Bars present as mean ± S.E.M. of percent analgesia for group of six animals. The data was analyzed by ANOVA followed by Dunnett's test. Asterisks indicated statistically significant values from control. *P < 0.05, **P < 0.01.

The results showed that the pain relief was achieved in a dose dependent manner, at all test doses (100, 200 and 300mg/kg i.p.) as shown in Table 2. Maximum inhibition (78.90%) was observed at 300mg/kg dose of VBME. The percent inhibition of writhing is shown in Figure 2. The inhibitory effect of paracetamol (96.22%) was greater than that of the highest dose of VBME.

**Table 2:** Effect of VBME 100, 200 and 300mg/kg in acetic acid induced test

**Treatment**	**Dose (mg/kgi.p.)**	**No. of writhing (10min)**
Saline	10ml/kg	64.80 ± 2.68
Methanolic extract	100	38.80 ± 2.50**
	200	28.00 ± 1.50**
	300	20.00 ± 1.14**
Diclofenac	10	10.40 ± 1.36**

Values are reported as mean ± S.E.M. for group of six animals. The data was analyzed by ANOVA followed by Dunnett’s test. Asterisks indicated statistically significant values from control. *P < 0.05, **P < 0.01.

#### Hot plat test

**Figure 3:**
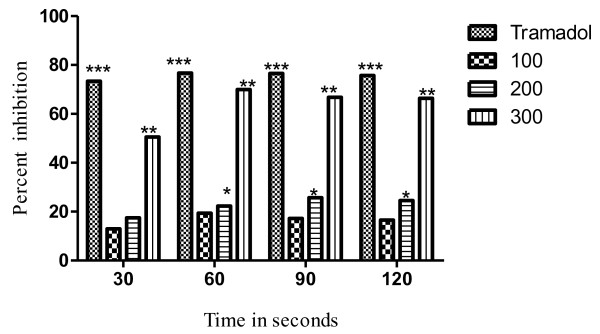
**Percent effect of VBME (100, 200 and 300mg/kg) and Tramadol**^**R**^**(30mg/kg) on hot plate pain in mice.** Each point represents the mean ± SEM of 6 animals, The data was analyzed by ANOVA followed by Dunnett’s test. Asterisks indicated statistically significant values from control. *P < 0.05, **P < 0.01, **P < 0.001.

**Figure 4:**
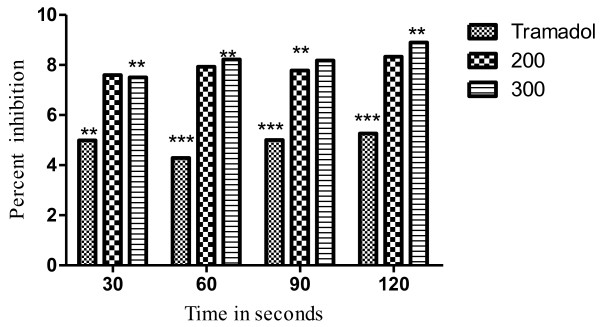
**Percent effect of VBME (200 and 300mg/kg) and Tramadol**^**R**^**(30mg/kg) antagonized with naloxone (0.5mg/kg) on hot plat pain model in mice.** Each point represent the mean ± SEM of six animals. The data was analyzed by ANOVA followed by Dunnett’s test. Asterisks indicated statistically significant values from control. *P < 0.05, **P < 0.01, **P < 0.001.

The results of the hot plat test revealed that the latency time was significantly (*P <* 0.05) increased from 17.22% to 68.58% at the dose of 100 to 300mg/kg. The effect was dose dependent and the maximum effect was observed after 60min as shown in Table 3. The most significant (*P* < 0.01) increase in latency time noticed against 300mg/kg of VBME was 68.58% whereas, the percent inhibition of the standard opioid analgesic (Tramadol^R^) was 76.73% as shown in Figure 3. In the presence of naloxone, the analgesic effect of Tramadol^R^ (30mg/kg) and VBME (200 and 300mg/kg) was reversed profoundly (Figure 4).

**Table 3:** Effect of VBME 100, 200 and 300mg/kg in hot plat test

**Group**	**Treatment/kg**	**0min**	**30min**	**60min**	**90min**	**120min**
Saline	10ml	9.20 ± 0.02	9.22 ± 0.08	9.16 ± 0.09	9.20 ± 0.03	9.12 ± 0.11
VBME	100mg	9.21 ±0.23	11.92 ± 0.44	13.20* ± 0.87	12.78* ± 0.46	12.58* ± 0.34
	200mg	9.23 ±0.65	12.85 ± 0.87	14.51** ± 0.54	14.30** ± 0.84	14.09** ± 0.91
	300mg	9.24 ±0.76	19.76* ± 0.22	23.74** ± 0.12	23.10** ± 0.12	22.98** ± 0.69
Tramadol^R^	30mg	9.20 ±0.02	25.34** ± 0.04	25.88*** ± 0.06	25.80*** ± 0.07	25.77*** ± 0.00
Analgesic effect of Tramadol^R^ and VBME antagonized by Naloxone						
VBME	300mg	9.21 ±0.45	10.78** ± 0.56	10.80** ± 0.39	10.91** ± 0.89	10.98** ± 0.78
	200mg	9.22 ±0.76	10.80** ± 0.87	10.75** ± 0.92	10.82** ± 0.72	10.86** ± 0.92
Tramadol^R^	30mg	9.23 ±0.02	10.22** ± 0.05	10.02*** ± 0.09	10.24*** ± 0.03	10.05*** ± 0.00

Values are reported as mean ± S.E.M. for group of six animals. The data was analyzed by ANOVA followed by Dunnett’s test. Asterisks indicated statistically significant values from control. *P < 0.05, **P < 0.01, ***P < 0.001.

#### Tail immersion test

**Figure 5:**
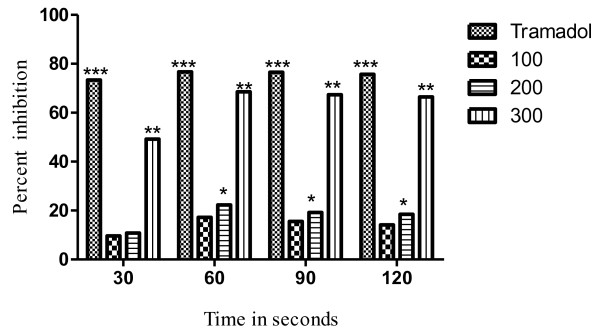
**Percent effect of VBME (100, 200 and 300mg/kg) and Tramadol**^**R**^**(30mg/kg) on tail immersion pain in mice.** Each point represents the mean ± SEM of 6 animals. The data was analyzed by ANOVA followed by Dunnett’s test. Asterisks indicated statistically significant values from control. *P < 0.05, **P < 0.01, ***P < 0.001.

The analgesic effect of the VBME was also significant (*P <* 0.05) in tail immersion test and was dose dependent like hot plat test. The reaction time of all doses and Tramadol^R^ is given in Table 4. The maximum analgesic effect was noticed at 60min after the dose administration. The percent inhibition of pain was 17.22, 22.29 and 68.58 at 100, 200 and 300mg/kg of VBME respectively. Tramadol^R^ which is a centrally acting opioid analgesic showed marked activity (76.73%) as shown in Figure 5.

**Table 4:** Effect of VBME 100, 200 and 300mg/kg in Tail immersion test

**Group**	**Per Kg**	**0min**	**30min**	**60min**	**90min**	**120min**
Saline	10ml	3.22 ± 0.02	3.23 ± 0.04	3.31 ± 0.04	3.28 ± 0.10	3.25 ± 0.12
VBME	100	3.21 ± 0.87	3.54 ± 0.28	3.88* ± 0.28	3.79 * ± 0.67	3.71* ± 0.24
	200	3.22 ± 0.28	3.58* ± 0.18	3.95** ± 0.23	3.91** ± 0.48	3.85 ** ± 0.97
	300	3.25 ± 0.27	4.82* ± 0.22	5.58** ± 0.72	5.49 ** ± 0.76	5.41** ± 0.27
Tramadol^R^	30mg	3.20 ± 0.01	5.60*** ± 0.03	5.85*** ± 0.03	5.79*** ± 0.08	5.71*** ± 0.00

Values are reported as mean ± S.E.M. for group of six animals. The data was analyzed by ANOVA followed by Dunnett’s test. Asterisks indicated statistically significant values from control. *P < 0.05, **P < 0.01, ***P < 0.001.

### Anti-inflammatory activity

#### Edema induced by Carrageenan

**Figure 6:**
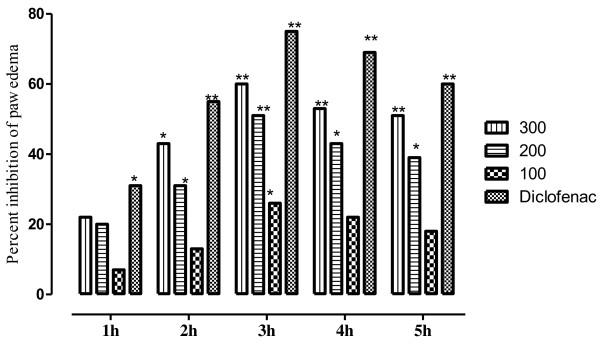
**Anti-inflammatory effect of VBME (100, 200 and 300mg/kg) in carrageenan induced paw edema in mice.** Each point percent inhibition of paw edema after 1,2,3,4 and 5h of treatment. The data was analyzed by ANOVA followed by Dunnett’s test. Asterisks indicated statistically significant values from control. *P < 0.05, **P < 0.01.

The anti-inflammatory activity at test doses (100, 200 and 300mg/kg i.p.) of VBME is presented in Table 5 with the average volume of the paw edema. The percent protection of inflammation is presented in Figure 6. The injection of the carrageenan in paw created an inflammatory edema which increased gradually. The VBME at the dose of 300mg/kg exhibited an anti-inflammatory activity that became significant (*P* < 0.01) 2 h after the injection of carrageenan and was maintained all along the experiment with a maximum effect of 60.880%. The extract (200 and 300mg/kg) induced significant (*P* < 0.01) anti-inflammatory effect and the anti-inflammatory effect of diclofenac sodium (10mg/kg) was greater than that of the extract as presented in Figure 6.

**Table 5:** Effect of intraperitoneal administration of VBME at 100, 200 and 300mg/kg in carrageenan and histamine induced paw edema test

**Treatment**	**Dose mg/kg**	**NPS**	**0h**	**1h**	**2h**	**3h**	**4h**	**5h**
Saline	10ml	0.0960 ± 0.22	0.2160 ± 0.24	0.2180 ± 0.12	0.2195 ± 0.20	0.2040 ± 0.14	0.2040 ± 0.18	0.2060 ± 0.17
Diclofenac	10mg	0.0900 ± 0.20	0.2180 ± 0.23	0.1487* ± 0.02	0.0980** ± 0.08	0.0499** ± 0.03	0.0620** ± 0.02	0.0821** ± 0.06
**Anti-inflammatory effect against carrageenan induced paw edema**								
VBME	100	0.0920 ± 0.24	0.2140 ± 0.20	0.2020 ± 0.09	0.1902 ± 0.16	0.1499* ± 0.35	0.1580 ± 0.14	0.1680 ± 0.24
	200	0.0980 ± 0.18	0.2060 ± 0.19	0.1723* ± 0.27	0.1500* ± 0.29	0.0998** ± 0.31	0.1145* ± 0.40	0.1240* ± 0.20
	300	0.0980 ± 0.20	0.2190 ± 0.22	0.1680* ± 0.27	0.1247* ± 0.32	0.0799** ± 0.19	0.0940** ± 0.37	0.1009** ± 0.17
**Anti-inflammatory effect against histamine induced paw edema**								
VBME	200	0.0970 ± 0.12	0.2070 ± 0.42	0.1628* ± 0.43	0.1520* ± 0.21	0.0999** ± 0.22	0.1091* ± 0.31	0.1109* ± 0.19
	300	0.0967 ± 0.32	0.2092 ± 0.53	0.1185* ± 0.87	0.1155* ± 0.24	0.0810** ± 0.71	0.0905** ± 0.33	0.1013** ± 0.18

NPS (Normal Paw size). Values are reported as mean ± S.E.M. for group of six animals. The data was analyzed by ANOVA followed by Dunnett’s test. Asterisks indicated statistically significant values from control. *P < 0.05, **P < 0.01.

#### Histamine induced paw edema

**Figure 7:**
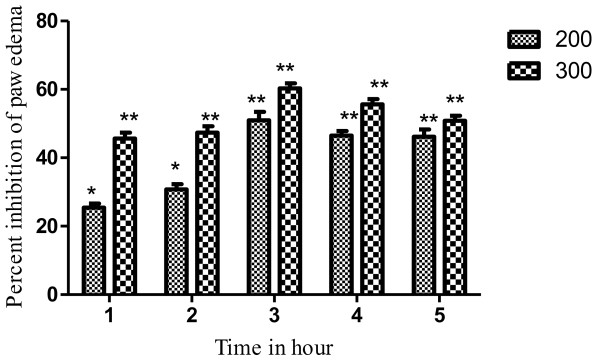
**Anti-inflammatory effect of VBME (200 and 300mg/kg) in histamine induced paw oedema model in mice.** Bar presents the percent inhibition of paw oedema after 1,2,3,4 and 5h of the treatment. The data was analyzed by ANOVA followed by Dunnett’s test. Asterisks indicated statistically significant values from control. *P < 0.05, **P < 0.01.

The inflammatory edema induced by histamine was significantly inhibited by VBME 200 and 300mg/kg. The VBME showed a reasonable anti-inflammatory effect in a dose dependent manner and remained significant up to 5^th^ h of administration as shown in Table 5 and Figure 7.

#### Phytochemical test

The VBME was found to contain alkaloids, saponins, flavonoids, tannins, proteins, and phenolic compounds.

## Discussion

Results of the present study showed that the VBME has marked antipyretic, analgesic and anti-inflammatory effects with a reasonable safety profile.

Subcutaneous injection of Brewer’s yeast induces pyrexia by increasing the synthesis of prostaglandin. It is considered as a useful test for the screening of plants materials as well as synthetic drugs for their antipyretic effect [14,18]. Yeast-induced pyrexia is called pathogenic fever and its etiology could be the production of prostaglandins [19]. The inhibition of prostaglandin synthesis could be the possible mechanism of antipyretic action as that of paracetamol and the inhibition of prostaglandin can be achieved by blocking the cyclo-oxygenase enzyme activity. There are several mediators for pyrexia and the inhibition of these mediators are responsible for the antipyretic effect [20]. The intraperitoneal administration of VBME significantly attenuated rectal temperature of yeast induced febrile mice. Thus it can be postulated that VBME contained pharmacologically active principle(s) that interfere with the release of prostaglandins. Furthermore, the presence of salicylic acids in the other species of the genus *Viola*[21] and the antipyretic action of the *n-*hexane fraction of *Viola odorata*[22] supplement the antipyretic activity of our tested extract.

Acetic acid-induced writhing is a well recommended protocol in evaluating medicinal agents for their analgesic property. The pain induction caused by liberating endogenous substances as well as some other pain mediators such as arachidonic acid via cyclooxygenase, and prostaglandin biosynthesis [10,23]. This pain paradigm is widely used for the assessment of peripheral analgesic activity due to its sensitivity and response to the compounds at a dose which is not effective in other methods. The local peritoneal receptor could be the cause of abdominal writhings [24]. Pain sensation in acetic acid induced writhing paradigm is elicited by producing localized inflammatory response due to release of free arachidonic acid from tissue phospholipids via cyclo-oxygenase (COX), and producing prostaglandin specifically PGE2 and PGF2α, the level of lipoxygenase products may also increases in peritoneal fluids [10,23]. These prostaglandin and lipoxygenase products cause inflammation and pain by increasing capillary permeability. The substance inhibiting the writhings will have analgesic effect preferably by inhibition of prostaglandin synthesis, a peripheral mechanism of pain inhibition [23]. Regarding the results of our extract in acetic acid-induced abdominal constriction assay, a prominent inhibition of writhing reflux was observed. These findings strongly recommend that VBME has peripheral analgesic activity and their mechanisms of action may be mediated through inhibition of local peritoneal receptors which may be the involvement of cyclooxygenase inhibition potential. The profound analgesic activity of VBME may be due to the interference of their active principle(s) with the release of pain mediators.

Thermal nociception models such as hot plat and the tail immersion tests were used to evaluate central analgesic activity. VBME showed significant (*P* < 0.01) analgesic effect in both the hot plat and tail immersion tests, implicating both spinal and supraspinal analgesic pathways. In these pain paradigms Tramadol^R^, which is similar to the action of opioid agonists (e.g. morphine), raised the pain threshold level within 30min of administration. In contrast, VBME showed maximum analgesic effect after 60min of administration. This difference in the maximum analgesic point could be explained by difference in the metabolic rate of each drug or may be the potency of each drug as the analgesic potential of Tramadol^R^ is higher than VBME (300mg/kg). Moreover, VBME showed a maximum effect after 60min and remain up to 120min in both thermal tests. When the nonselective opioid receptor antagonist naloxone was applied, the analgesic effect of VBME was also antagonized by naloxone after 30min of administration; it means that the analgesic effect of this extract is due to activation of the opioid receptor stimulation.

Carrageenan-induced paw edema is a well established animal model to assess the anti-inflammatory effect of natural products as well as synthetic chemical compounds. Edema formation due to carrageenan in paw is a biphasic event, during 1–5h; the initial phase (1h or 1.5h) is predominately a non-phagocytic edema followed by a second phase (2–5) h with increased edema formation that remained up to 5h [14,25]. The initial phase has been induced due to the action of mediators such as histamine, serotonin and bradykinin on vascular permeability [26]. The late phase or second phase edema has been shown to be the result of overproduction of prostaglandins [27]. The result of pre-treatment of VBME demonstrated that the extract (200 and 300mg/kg i.p.) is effective in the early phase of inflammation which is due to release of histamine and serotonin primarily. The anti-inflammatory effect of the extract remains significant up to 5^th^ h of the experiment. VBME showed significant activity against histamine induce edema in both phases. During preliminary phytochemical screening of the crude extract, important therapeutic principles like alkaloids, saponins, flavonoids, tannins etc. were detected. Therefore, the current findings can be attributed to these groups of chemical compounds. Further study is need on VBME to find the exact mechanism of action for its antipyretic, analgesic and anti-inflammatory effects.

## Conclusion

In conclusion, the methanolic extract of *Viola betonicifolia* was proved a natural safe remedy for the treatment of pyrexia, algesia and inflammation. Our current findings demonstrated scientific rationale for the folk use of the plant as antipyretic, analgesic and anti-inflammatory. Interestingly the VBME exhibited both peripheral as well as central analgesic effect which might have been attributed to the presence of such active principles, due to which it has proven folk use in various nervous disorders. Nevertheless, the isolation of pure secondary metabolites from the plant will help us further in understanding the mechanism of these activities and identification of lead compounds of clinical utility.

## Competing interest

The authors declare that they have no competing interests.

## Author’s contribution

Muhammad Saeed was project supervisor and proof read the manuscript. Naveed Muhammad carried out experimental work and Haroon Khan was involved in project designing and writing of manuscript. All authors read and approved the final manuscript.

## Pre-publication history

The pre-publication history for this paper can be accessed here:

http://www.biomedcentral.com/1472-6882/12/59/prepub
